# Robot-Assisted Surgery vs Robotic Stereotactic Body Radiotherapy in Prostate Cancer: A Cost-Utility Analysis

**DOI:** 10.3389/fonc.2022.834023

**Published:** 2022-05-24

**Authors:** Line Farah, Nicolas Magne, Nicolas Martelli, Sandrine Sotton, Marc Zerbib, Isabelle Borget, Nathaniel Scher, Thierry Guetta, Cyrus Chargari, Olivier Bauduceau, Alain Toledano

**Affiliations:** ^1^ Groupe de Recherche et d’accueil en Droit et Economie de la Santé (GRADES) Department, Université Paris Saclay, Châtenay-Malabry, France; ^2^ Department of the Innovation Center for Medical Devices, Innovation Center for Medical Devices (CiDM), Hôpital Foch, Suresnes, France; ^3^ Département de radiothérapie, Institut de Cancérologie Lucien Neuwirth, Saint Priest en Jarez, France; ^4^ Département de pharmacie , Hôpital Européen Georges Pompidou (HEGP), Paris, France; ^5^ Département d’urologie , Service d’urologie, Hôpital Cochin, Paris, France; ^6^ Département d’études en recherche et économie, Institut Gustave Roussy, Villejuif, France; ^7^ Département d’économie de la santé, Institut National de la Santé et de la Recherche Médicale (INSERM), Paris, France; ^8^ Département de radiothérapie, Institut de radiothérapie et de radiochirurgie H. Hartmann, Paris, France; ^9^ Département d’urologie, Clinique Ambroise Paré, Neuilly-sur-Seine, France; ^10^ Département d’oncologie en radiothérapie, Institut Gustave Roussy, Villejuif, France

**Keywords:** robot-assisted radical prostatectomy, prostate cancer, health economic analysis, quality of life, cost-utility analyses, stereotactic body radiation therapy (SBRT)

## Abstract

Prostate cancer is the most common men cancer in France. Continuous progress in oncology led to develop robot-assisted Radical Prostatectomies (rRP) and robot-assisted stereotactic body radiotherapy (rSBRT). The present study aims at comparing economic and clinical impacts of prostate cancer treatments performed either with rSBRT or rRP in France. A Markov model using TreeAge Pro software was chosen to calculate annual costs; utilities and transition probabilities of localized prostate cancer treatments. Patients were eligible for radiotherapy or surgery and the therapeutic decision was a robot-assisted intervention. Over a 10-year period, rSBRT yielded a significantly higher number of quality-adjusted life years than rRP (8.37 vs 6.85). In France, rSBRT seemed more expensive than rRP (€19,475 vs €18,968, respectively). From a societal perspective, rRP was more cost-saving (incremental cost effectiveness ratio = €332/QALY). The model was sensitive to variations of costs of the initial and recurrence state in one-way sensitivity analyses. Robot-assisted stereotactic body radiotherapy seems more cost-effective than Radical Prostatectomy in terms of QALY despite the slightly higher initial cost due to the use of radiotherapy. It would be interesting to conduct comparative quality of life studies in France over longer periods of time.

## Introduction

Despite significant progress in early detection of prostate cancer, it remains the first leading cause of male cancer death in France with 8,512 deaths annually (1). It is responsible for nearly a quarter of all cancers and more than 50,430 new cases diagnosed yearly in France (1). The development of new surgical techniques and medical devices has offered new possibilities to treat this pathology.

The main therapeutic modalities for treating localized prostate cancer are external radiation treatments such as Intensity-Modulated Radiation Therapy (IMRT) as well as brachytherapy (or a combination of both) and surgery (radical prostatectomy). Stereotactic radiotherapy is one of the therapeutic standards recommended by the American Society for Radiation Oncology (ASTRO) (2) and the National Comprehensive Cancer Network (NCCN) (3) but, it has not yet been included in French guidelines. Some surgical teams have chosen robot-assisted surgery as a standard operating technique for localized prostate cancer. The surgeon still removes the prostate and the seminal vesicles but the intervention is enhanced by robotics (4). In parallel to these minimally invasive robot-assisted Radical Prostatectomy (rRP) procedures, there have also been recent advances in radiotherapy (5). The latest key innovation is the development of robot-assisted stereotactic body radiotherapy (rSBRT), with Cyberknife™ robot (Accuray) for instance. This non-invasive irradiation technique delivers a high dose to a small volume (6). The low toxicity of rSBRT and its capacity to improve quality of life make it at least comparable and as well tolerated as other radiotherapy techniques (such as proton therapy, brachytherapy or Intensity-modulated radiotherapy) (5). rSBRT is also an effective option for the elderly or to patients in whom surgery is contraindicated. To date, the economic and societal benefits of SBRT performed by Cyberknife still require a more extensive assessment over longer follow-up periods. In the USA, studies has shown that rSBRT is considered more cost-effective than IMRT (7). Reducing treatment duration would mean an improvement of patients’ quality of life and a reduction in treatment costs (e.g. lower ambulance transportation costs). Moreover, robotic radiotherapy with Cyberknife uses artificial intelligence to localize tumours.

However, these different robot-assisted therapies have not been compared and the current European and French guidelines regarding low-risk localized prostate cancer (as defined by the D’Amico classification) do not favour one type of intervention over the other, even though localized prostate cancers represent 40 to 50% of all prostate cancers diagnosed in France (1).

Furthermore, surgical robots implemented in French operating theatres do not require any specific authorizations. As a result, the costs of a robot are negotiated by each hospital and are not covered by the French national health insurance scheme. Robot-assisted radical prostatectomies accounted for 73% of 20,380 procedures performed in France in 2018 (8). There is also a general lack of economic data to substantiate the additional costs and potential benefits of this technique. Consequently, an economic evaluation comparing these new therapies, in particular rRP and rSBRT, would permit to estimate their potential benefits for patients (at a clinical level) and institutions (at an economical level) and thereby, provide a tool to assist financial decision-makers.

## Materials and Methods

The current cost-utility analysis sought to understand the economic and long-term clinical impacts of treating prostate cancer with rSBRT rather than rRP, in France. In order to build our health economic model to compare both strategies, we created a Markov model structure with four states. For each state, we determined clinical inputs, quality of life relative to utilities and costs inputs. Finally, sensitivity analysis was performed to assess uncertainty of model parameters and robustness of the model.

### Study Design

A Markov cost-effectiveness model was developed to compare incremental costs and quality-adjusted life years (QALYs) of rSBRT/rRP. The analysis was conducted from a societal perspective over a 10-year time horizon. It included costs related to interventions, side effects (affecting sexual, bowel and urinary functions as well as bleeding), medical visits, transportation and follow-up. The article was written according to the ISPOR CHEERS checklist (9).

### The Markov Model Structure

**Figure 1:**
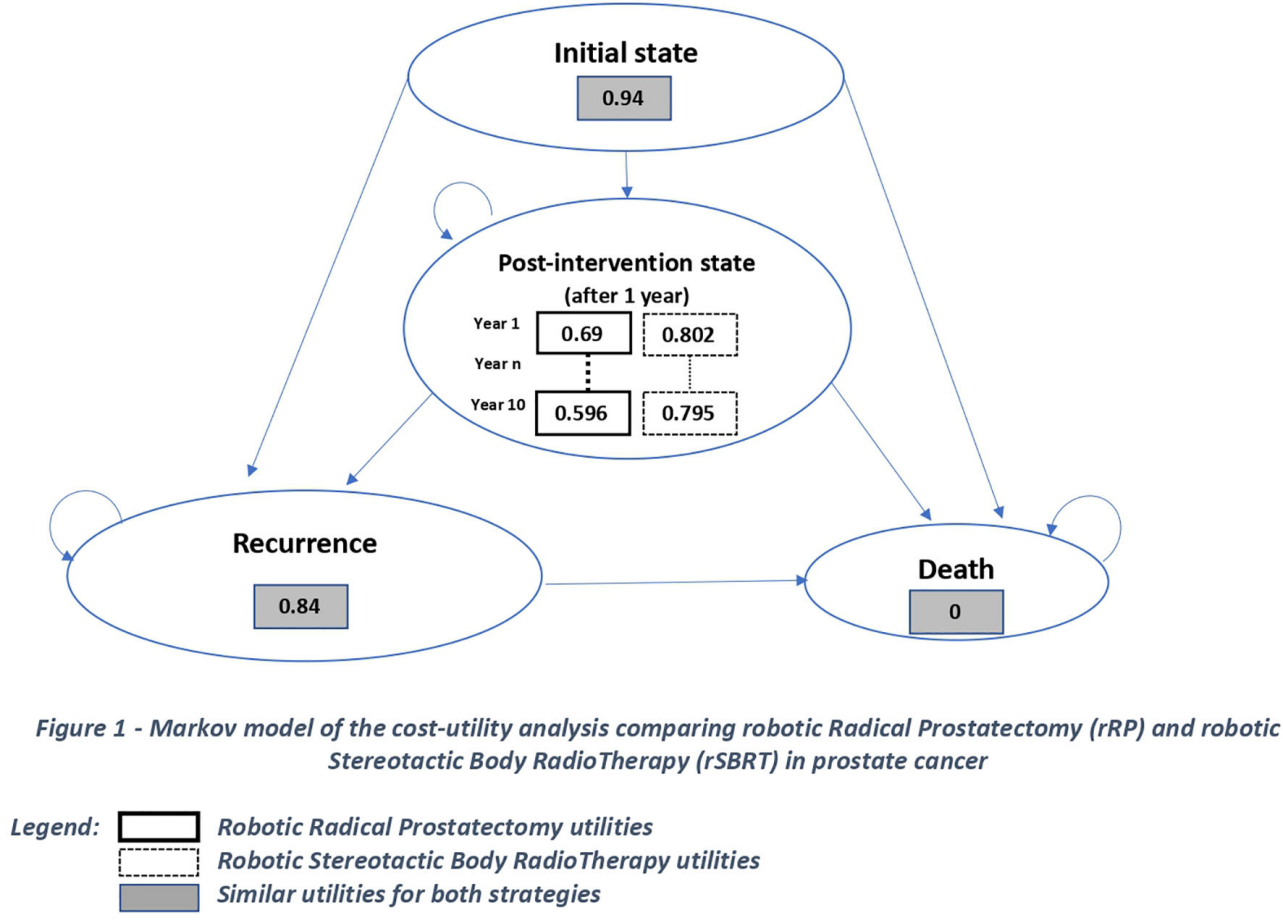
Markov model of the cost-utility analysis comparing rRP and rSBRT in prostate cancer.

Our model included two treatment strategies: robot-assisted radical prostatectomy and robotic stereotactic body radiotherapy. The model was constructed using 1-year cycles and estimated cost effectiveness for a period of up to 10 years. Within each cycle, patients could experience clinical events leading to recurrence or death and associated costs and quality of life (QoL) adjustments (**Figure 1**). The model differentiated four distinct states as recommended by the radiotherapeutic oncology team. An “Initial state” (patient’s condition between the intervention and the first year following the intervention), a “1-year post-interventional state” (patient’s condition after the first year of treatment) -added to take into account the lack of memory in a Markov model- a “recurrence” status (detected during routine follow-up, which could require surgery, radiation therapy or drug interventions or an increased number of follow-up consultations) and “Death”.

One of our hypotheses was that over the 10-year time horizon of our model, the state of “distant metastasis” would not be modeled. Our model used the TreeAge Pro software (v2022). We calculated the annual costs *per* patient for each of the two treatment strategies and the utilities and transition probabilities between each state.

### Clinical Inputs and Quality of Life

A team composed of radiation oncologists and urologists defined the target population. Low-risk localized (non-metastatic) prostate cancer cases as defined by the D’Amico classification (intracapsular cancer (T1 or T2a), PSA <10 and Gleason score <7) were included [3]. The therapeutic decision discussed at a urology tumor board was a robot-assisted intervention (by surgery or by stereotactic radiotherapy). Patients were eligible for radiotherapy or surgery.

Within the model, we determined the probabilities of a transition between the different states as well as the costs and the utilities of each state (**Table 1**) (13). Individual parameter values were determined from a literature review performed on 08/01/2019 without period specification (Medline) and from medical experts’ interviews. Articles selection and the flow chart are detailed in the **Supplementary File**. Utility values for each state were reported in the literature for prostate cancer patients (**Table 1**). The utilities, *i.e.* the units that estimate the quality of life, were found in the studies selected in our literature review. In the current analysis, utility values were compared to the corresponding baseline values obtained for prostate cancer patients prior to rRP or rSBRT (**Table 1**). The average utilities of the “post-intervention” state (**Table 1**) was estimated based on utility data reported in the literature and the patients’ likelihood to experience sexual, urinary and bowel dysfunctions, compared to the baseline utility values established at the time the patient was included in the study (7). Utility values highlighted some aspects of quality of life that were elaborated in the prostate cancer specific quality of life questionnaires (Expanded Prostate Cancer Index Composite). Depending on the type of intervention, we compared values ​​from the Katz et al. study (5), the PACE-B study (10) and the PROTECT trial (11) and the probability of occurrence of any adverse effects to calculate the average utility *per* year (5). Utility values decreased after intervention because they captured the decrement in QoL due to age but also due to the burden of prostate cancer, while the probability of death increased over the 10-year period. This decreasing utility values were consistent with the age-related decline of the general population reported in the French INSEE database (14).

**Table 1:** Summary of utilities, costs and transition probabilities of the different states calculated and collated for rRP and rSBRT.

UTILITIES & COSTS							
		rSBRT - robotic Stereotactic Body RadioTherapy		rRP - robotic Radical Prostatectomy			
States		Costs (€)	Utilities	Costs (€)		Utilities	Sources
**Initial**		€ 10,815	0.94 (CI_95 =_ 0.90-0.98)	€ 8,881		0.94 (CI_95 =_ 0.90-0.98)	Utility (5): Costs: details about initial costs calculation in table 2 of the Supplementary material B
**Post-intervention » (after 1 year)**	**Year 1**	€ 902	0.802 (CI_95 =_ 0.752-0.852)	€ 902		0.69 (CI_95 =_ 0.64-0.74)	Utility : Katz et al., PACE-B, PROTECT (5), (10), (11) Cost (12):
	**Year 2**		0.789 (CI_95 =_ 0.739-0.839)			0.687 (CI_95 =_ 0.637-0.737)	
	**Year 3**		0.795 (CI_95 =_ 0.745-0.845)			0.719 (CI_95 =_ 0.669-0.769)	
	**Year 4**		0.795 (CI_95 =_ 0.745-0.845)			0.594 (CI_95 =_ 0.476-0.714)	
	**Year 5**		0.795 (CI_95 =_ 0.745-0.845)			0.603 (CI_95 =_ 0.483-0.724)	
	**Year 6+**		0.795 (CI_95 =_ 0.745-0.845)			0.596 (CI_95 =_ 0.477-0.715)	
**Recurrence**		€ 13,707	0.84 (CI_95 =_ 0.81-0.87)	€ 13,707		0.84 (CI_95 =_ 0.79-0.89)	Utility (5): Cost (12):
**TRANSITION PROBABILITIES**							
**Transition**	** **	**Probabilities (%)**	**Sources**	**Probabilities (%)**	**Sources**		
**Transition probability from «initial state» to « recurrence »**	** **	0.0011	[7]	0.00255	[20] + [10]		
**Transition probability from « post-intervention » state (after 1 year) to « Recurrence »**		0.0011	[7]	0.00255	[20] + [10]		
**Transition probability from «initial state » to «Death»**	** **	0.00979	[11]	0.00979	[11]		

Quality-adjusted life years (QALYs) were calculated by multiplying the length of time in a state by the utility for the given state. QALYs were discounted at an annual rate of 4% as recommended by the French Health Agency (15).

### Costs Inputs

The analysis considered direct costs as well as costs associated with long-term disability care provided in facilities. Cost data was collated from multiple sources including the French Diagnosis Related Group (DRG) system for 2021 and published costs (**Table 1**) (12, 16). The cost data used in our model refers to French national data. Indeed, the DRGs correspond to the price of a hospitalization for prostatectomy or radiotherapy session in France (regardless of the type of hospital). The details of the calculations specified in our **Supplementary File** correspond to the average national costs of prostate cancer treatment in France.

The “Initial state” costs were calculated from French databases for each type of intervention (**Supplementary File**) (16–21). All costs were in Euros for the year 2021. Future costs were discounted at an annual rate of 4% as recommended by the French Health Agency (15).

### Sensitivity Analysis

Uncertainty of model parameters was assessed using one-way deterministic analysis and probabilistic sensitivity analyses. Treatment-specific inputs included all transition probabilities, costs and health utilities.

One-way sensitivity analysis assessed the impact on model outcomes from a variation of input parameters of -/+20% unless otherwise noted, which included 95% confidence intervals (CI). Probabilistic sensitivity analyses assessed the overall uncertainty in the values used in the model and were based on a Monte Carlo simulation of 1000 iterations of the model over a 10-year time frame. Results are reported as an incremental cost-effectiveness ratio (ICER).

Finally, we estimated the “willingness to pay” which is the estimate of the willingness of the French financial decision-maker, namely health insurance, to pay for an intervention rather than another. The patient does not pay for his treatment whose costs are covered 100% by the French health insurance. Therefore, the amount paid by health insurance was taken into account in the model.

## Results

Over a 10-year period, robotic stereotactic radiotherapy yielded a significantly higher number of QALYs than robot assisted radical prostatectomy (8.373 *vs* 6.845, respectively). However, in France, rSBRT seemed more expensive than rRP (€19,475 *vs* €18,968, respectively). This led to an incremental cost of €507 for rSBRT compared to rRP over a 10-year period (**Table 2**).

**Table 2:** Costs and QALYs (Quality Adjusted Life Years) differences between the two strategies (rRP versus rSBRT) in order to estimate the ICER (Incremental Cost Effectiveness Ratio) over a 10-year time horizon.

Strategy	Cost (€)	Incremental Cost (€)	QALY	Incremental QALY	ICER
robotic Radical Prostatectomy (rRP)	18,968		6.845		
robotic Stereotactic Body RadioTherapy (rSBRT)	19,475	507	8.373	1.528	332

**Figure 2:**
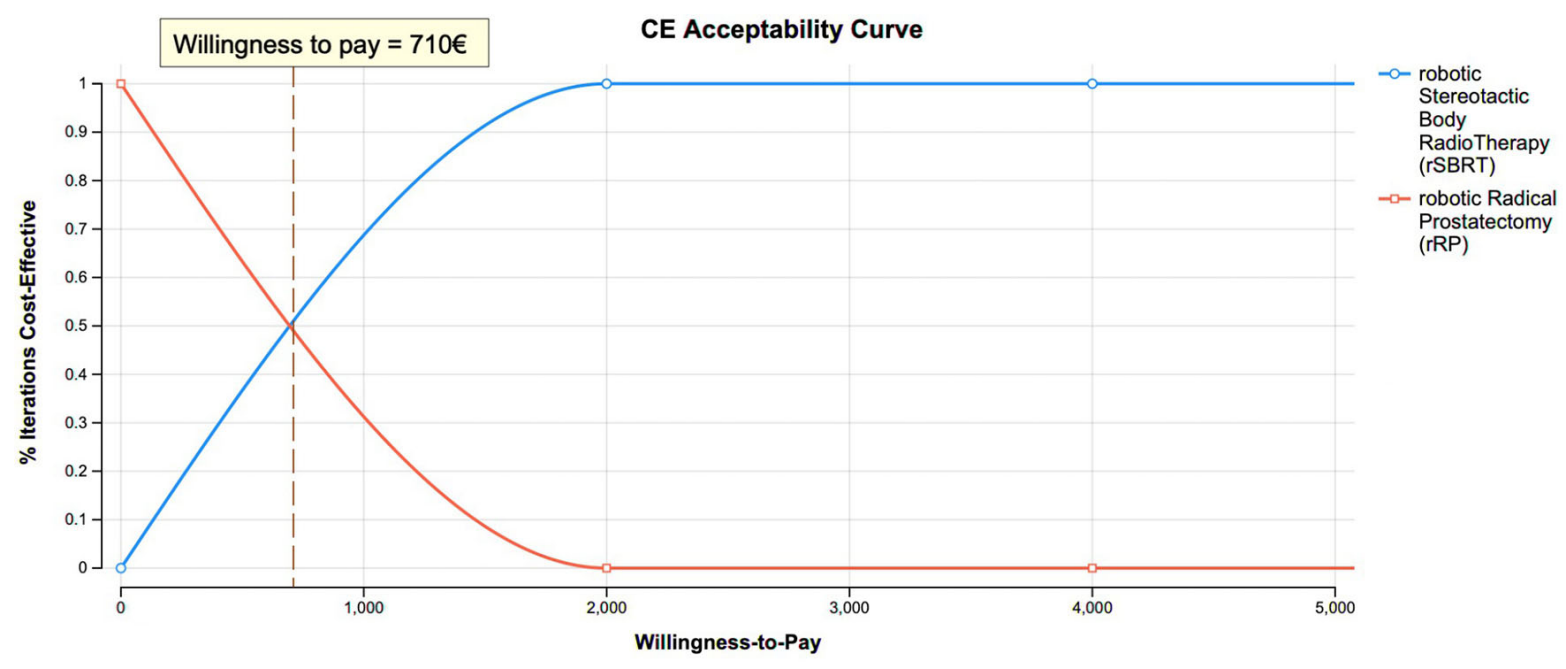
Acceptability Curve (rRP vs rSBRT).

From a societal perspective, rRP was cost saving when compared to rSBRT (ICER = €332/QALY over a 10-year time horizon). The acceptability curve (**Figure 2**) highlighted that, over a 10-year period, rSBRT became more cost-effective than rRP, beyond the €710 threshold (corresponding to the “willingness to pay”), from a societal perspective.

### One-Way Deterministic Sensitivity Analysis

**Figure 3:**
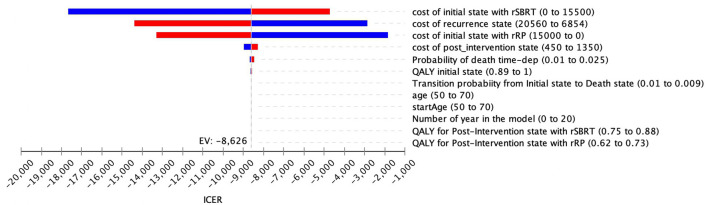
Tornado diagram (rSBRT vs RP): One-way sensitivity analysis and variation of the ICER as a function of parameters listed.

One-way sensitivity analyses, depicted in the Tornado diagram (**Figure 3**), illustrated that the model was more sensitive to cost variations of the initial state, regardless of the type of intervention (rRP/rSBRT) and to cost variations of recurrence state. The utilities values and the time horizon, entitled “number of years in the model”, had no significant impact on ICER. Therefore, the duration of the time horizon did not influence the results of our analysis.

### Probabilistic Sensitivity Analysis

**Figure 4:**
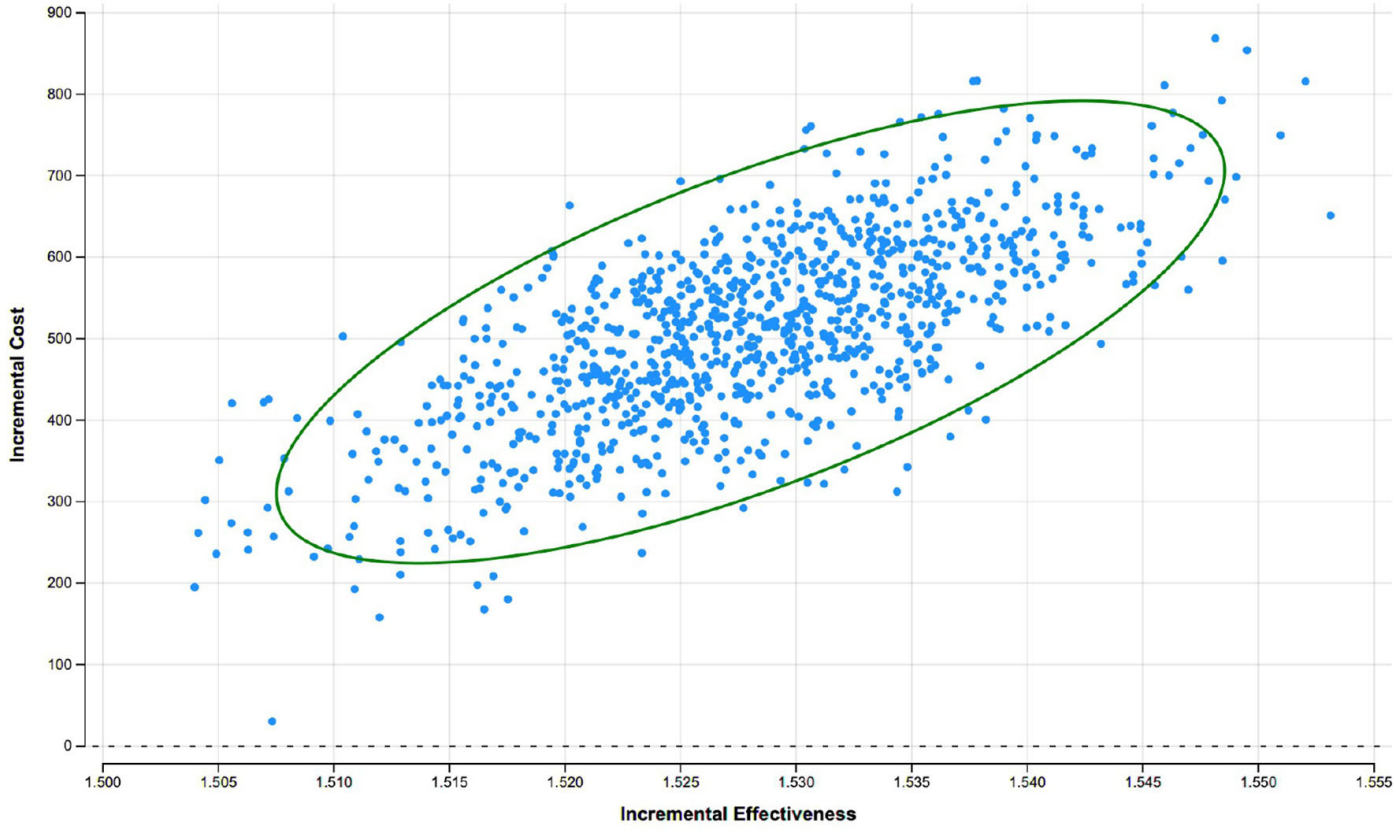
Cost-effectiveness plane rSBRT vs. rRP at 10 years: results of the probabilistic sensitivity analysis with a Monte Carlo simulation showing the dispersion of 1000 ICER.

Probabilistic sensitivity analyses -the dispersion of 1,000 ICER simulations- indicated that these ICERs were distributed in the northeast quadrant. The cost-effectiveness of rSBRT *vs* rRP was generally robust to changes in input variables. Dispersion is low. The incremental QALY values ​​range 1,51-1,56 and the incremental costs between €150 and €850 (**Figure 4**). In this scenario, robotic stereotactic radiotherapy is likely to be more effective, in terms of QALYs, and more expensive than robot-assisted radical prostatectomy over a 10-year time period.

## Discussion

**Figure 5:**
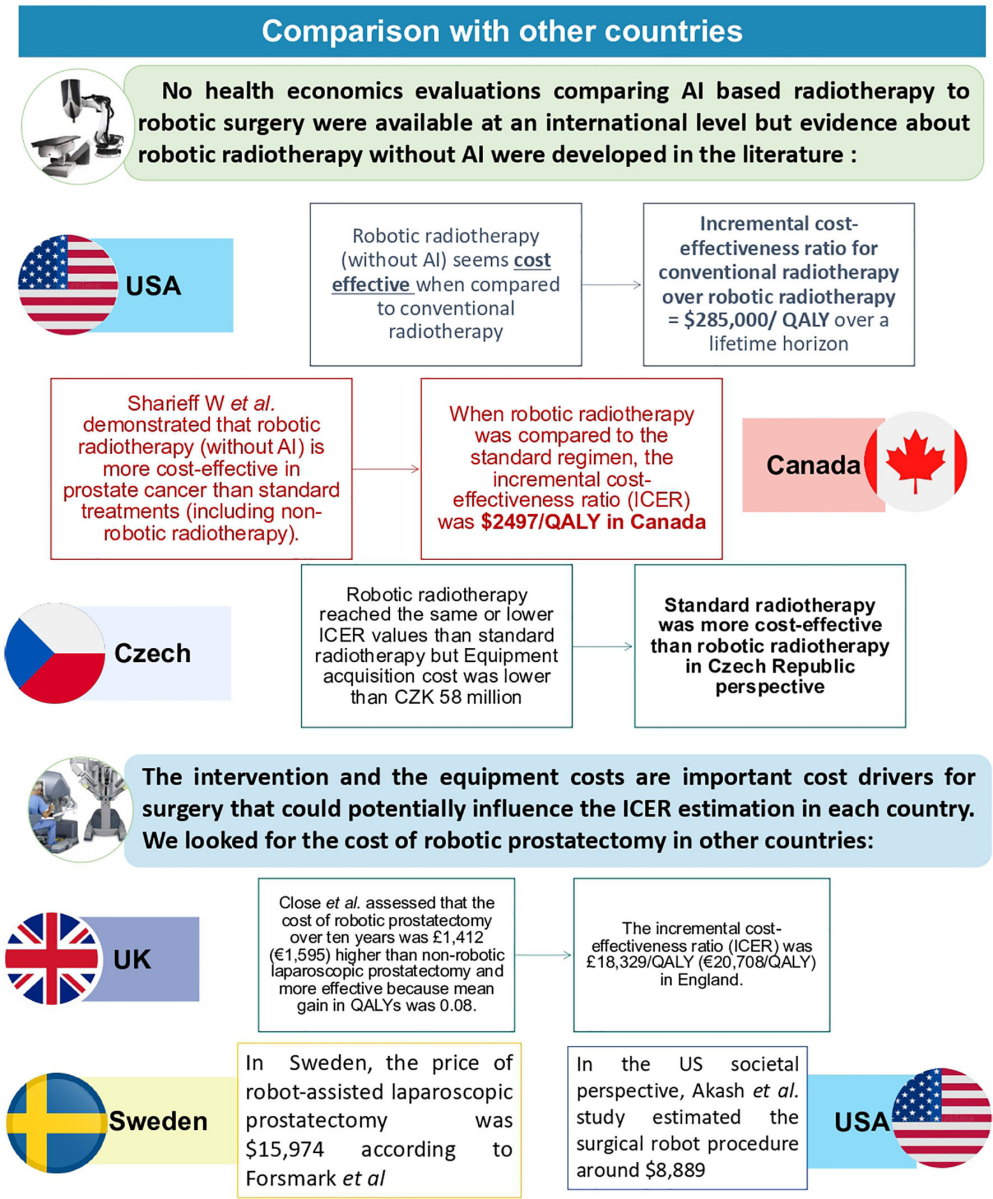
Comparison of rRP and rSBRT evaluations in other countries.

Based on data from the scientific literature and the estimated costs of treatments in France, our study suggests that, from a societal perspective, the use of rSBRT could prove cost-effective compared to rRP. Despite the moderate cost differential favoring rRP over a 10-year horizon (€507), rSBRT appeared to significantly improve patients’ quality of life (1.528 QALY corresponding to ICER €332/QALY). To our knowledge, this is the first economic evaluation that compares two robot-assisted curative robot-assisted interventions for the treatment of localized prostate cancer (rRP *vs* rSBRT). It is also the first economic evaluation that specifically addresses costs in France, unlike previous international studies (22). Our model is adapted to the French context but further studies should be conducted in other countries with suitable adaptations. This work could be repeated in another context to verify the generalization and robustness of these results. Even if rSBRT was not compared to rRP in previous studies, some studies focused the economic evaluation of rSBRT in comparison with intensity-modulated radiation therapy (IMRT) or proton therapy. In the United States, Sher et al. study concluded that robotic SBRT was more cost effective than conventional radiotherapy (IMRT) with an incremental cost-effectiveness ratio for conventional radiotherapy over robotic SBRT up to $285,000/QALY over a lifetime horizon for prostate cancer (7). Thus, rSBRT seems apparently less expensive but more toxic than conventional radiotherapy. In another American societal perspective, Parthan et al. evaluated that IMRT and proton therapy were both dominated by SBRT because they had higher costs and yielded fewer QALYs when compared with SBRT (ICERs: $9,991/-0.062 QALY for SBRT *vs* IMRT and $46,560/-0,047 QALY for SBRT *vs* PT) (23). In the Canadian societal perspective, Sharieff W et al. demonstrated that rSBRT was more cost-effective than standard treatments (including non-robotic SBRT) (24). When rSBRT was compared to the standard regimen using fixed-gantry system, the ICER was $2497/QALY for low-risk prostate cancer in Canada. Conversely, in the Czech healthcare system, rSBRT reached the same as/or lower ICER values than IMRT while the robotic SBRT acquisition cost was CZK 58 million lower. Therefore, IMRT was more cost-effective than rSBRT for localized prostate cancer treatment in Czech Republic perspective (25). We summarize in **Figure 5** the different outcomes of the previous mentioned countries related to their different healthcare financing systems.

In addition, the intervention and equipment costs are important cost drivers for surgery and they could potentially influence the ICER estimation in each country. Therefore, we looked for the cost of robotic prostatectomy or their health economic evaluation in other countries. In the United Kingdom, Close et al. assessed that the cost of robotic prostatectomy over ten years was £1,412 (€1,595) higher than non-robotic laparoscopic prostatectomy and more effective because mean gain in quality of life years was 0.08 (26). The incremental cost-effectiveness ratio (ICER) was £18,329/QALY (€20,708/QALY) in England. In the US societal perspective, Akash et al. estimated the surgical robot procedure around $8,889 (27). In Sweden, the price of robot-assisted laparoscopic prostatectomy was $15,974 according to Forsmark et al. (28). Finally, Perlbarg et al. literature review estimated the robot cost between €6,010 and €11,928 euros *per* patient in several countries (29). This difference suggested that further studies should be conducted in different countries in order to validate results.

However, to our knowledge, there have been no previous economic studies comparing robotic stereotactic radiotherapy/robot-assisted prostatectomy. In addition, we applied the recommendations of the French National Authority for Health to the model’s fundamental assumptions (evaluation method, target population, time horizon and updates) (15). We selected a cost-utility approach to evaluate localized prostate cancer because our patients’ 10-year life expectancy was the same as the 10-year life expectancy of same-age subjects from the general population. Thus, it is necessary to measure patients’ quality of life rather than merely assess specific patients’ survival end-points. We selected a 10-year follow-up period because a longer time frame would have unduly increased the degree of uncertainty of our results as clinical outcomes would not have been directly associated to any of the two interventions considered. A 10-year follow-up period permitted to evaluate the direct impact of the technique on patients’ outcome; which is less applicable beyond the 10-year period. The utility data used was derived from the Katz et al. study that evaluated medium-term quality of life (5). This type of analysis has the added advantage of taking into account the one-year short-term period. Moreover, it permits to model the progressive evolution of patients’ quality of life, 3 years after the intervention by integrating time-dependent QALY data.

As to costs, the cost of training of health care teams that, according to an interview conducted with experts (data not published), could amount to almost €800,000 in the case of rRP in public hospitals in Paris was not included. Costs of training staff for rSBRT should also be considered in the model but they are very difficult to document, as the Drummond et al. study showed (30). Training costs for rSBRT were not available this is why we could not integrate this parameter into our model. We based costs of the “recurrence” state on the Molinier et al. study (12). This estimate includes the costs of any secondary treatments within 5 years of the initial procedure and the total costs do not differ between the different disease risk levels (i.e., low, moderate or high risk). Since our current study exclusively focused on low-risk localized cancer cases, the assumptions based on the Molinier et al. study are expected to be conservative.

Our study includes several limitations. First, the absence of any prior study comparing rRP *versus* rSBRT in a French setting was problematic for the construction of our model. Indeed, it meant that we did not have any efficacy or quality of life data specific to French patients and further prospective studies about the French population are needed. Therefore, we assumed that the quality of life reported in the Katz et al. study for American patients would be similar for European ones. Secondly, our model did not consider a distal metastasis state since our target population consisted of low-risk patients followed up over a 10-year time horizon. Given the complexity of managing prostate cancer, we also had to simplify the treatment schedules in our model. We selected low-risk prostate cancer because the objective was to avoid adding confounding factors. If such analysis was chosen, we could not establish a direct correlation between the robot and its influence on costs or clinical results. Other variables could bias the analysis. Even if the two robotic techniques are among those that require higher financial investments because they are guided by robots, many therapeutic strategies in prostate cancer could be taken into account. Further health economic assessments such as Linac-based SBRT technology, for instance, could be particularly interesting. Finally, the active surveillance strategy initiated in patients whose cancer is not cured and who are classified in the “recurrence” state may potentially be confounded by additional psychological factors. We were unable to assess this impact from either a clinical or an economic perspective and therefore we omitted this state from the model. It would be interesting to consider an additional surveillance arm with real world data from further studies.

To conclude, there is an obvious lack of economic data to substantiate the additional costs and potential benefits of these different robot-assisted techniques. Thus, an economic evaluation comparing these new therapies, in particular robot-assisted radical prostatectomies and robot-assisted stereotactic body radiotherapy, would permit to estimate their benefits both for patients (at a clinical level) and for institutions (at an economical level). This would also provide a tool for financial-decision makers.

## Data Availability Statement

The original contributions presented in the study are included in the article/**Supplementary Material**. Further inquiries can be directed to the corresponding author.

## Author Contributions

LF, NMag, AT, NMar, IB, MZ, NS, TG, CC and OB contributed to the study conception and design. Material preparation, data collection and analysis were performed by LF, NMag, AT, NMar and IB. The first draft of the manuscript was written by LF, NMag, AT, NMar and IB. All authors commented on previous versions of the manuscript. All authors read and approved the final manuscript.

## Conflict of Interest

The authors declare that the research was conducted in the absence of any commercial or financial relationships that could be construed as a potential conflict of interest.

## Publisher’s Note

All claims expressed in this article are solely those of the authors and do not necessarily represent those of their affiliated organizations, or those of the publisher, the editors and the reviewers. Any product that may be evaluated in this article, or claim that may be made by its manufacturer, is not guaranteed or endorsed by the publisher.
