# Supplementary material for: Robot-Assisted Surgery vs Robotic Stereotactic Body Radiotherapy in Prostate Cancer: A Cost-Utility Analysis
Source: Front Oncol. 2022 May 24;12:834023. doi: 10.3389/fonc.2022.834023 (PMC9172203; doi:10.3389/fonc.2022.834023)
Supplement: Supplementary file 1 [file DataSheet_1.pdf]

Supplementary file

Study protocol

Decision model

A Markov model was selected for this cost-utility analysis because there is no interaction between patients (no competition to access available therapeutic alternatives). Moreover, in the population of interest, individuals have similar characteristics, so the cohort model is more suitable. In the natural history of the disease, the effects of treatments are long-lasting. The consideration of multiple recurring events is not possible with the decision trees (events take place at the same time) ; therefore, a Markov model is more accurate.

This model represents the possible transitions of a cohort of patients between multiple health conditions (or states) over several cycles that segment a time horizon. Health states are mutually exclusive, one of the states is absorbent (the death condition does not allow any transition to other states) and each of the states is associated with a cost and efficacy that may differ depending on the therapeutic alternative studied.

For each cycle, individuals can either remain in the same state of health or transit to another state. The probabilities of transition between states are determined by the state in which the patient is at the beginning of the cycle without taking into account the states through which he has previously passed. In localized prostate cancer, one-year cycles were determined, in agreement with a team of radiation oncologists.

We considered that patients with low-risk localized prostate cancer (according to D'Amico's classification) are individuals in general good condition for whom an intervention may be proposed. They have a life expectancy of at least 10 years. In our model, we decided to focus on a ten-year time horizon because, according to the results of a long-term RCT study published in the New England Journal of Medecine in 2016, « at a median of 10 years, prostate-cancer–specific mortality was low irrespective of the treatment assigned, with no significant difference among treatments. Surgery and radiotherapy were associated with lower incidences of disease progression and metastases than was active monitoring. » [1] We therefore hypothesized that, over the 10-year time horizon defined in our model, the state of "metastasis" was not be considered.

Data collection

The bibliographical research we conducted allowed us to identify the articles needed to calculate these parameters for patients with low-risk prostate cancer treated with prostatectomy or radiation therapy. The Medline database was interviewed in August 2019.

We used an advanced pubmed search algorithm (PubMed Advanced Search Builder) with the following MeSH Terms (Table 2). We also managed a manual article research in order to complete our foundings.

**Step 1**	((("prostatic neoplasms"[MeSH Terms] OR ("prostatic"[All Fields] AND "neoplasms"[All Fields]) OR "prostatic neoplasms"[All Fields] OR ("prostate"[All Fields] AND "cancer"[All Fields]) OR "prostate cancer"[All Fields]) OR ("prostatic neoplasms"[MeSH Terms] OR ("prostatic"[All Fields] AND "neoplasms"[All Fields]) OR "prostatic neoplasms"[All Fields])))
**Step 2**	AND ("prostatectomy"[MeSH Terms] OR "Prostatic Neoplasms/surgery"[MeSH Terms] OR Adenocarcinoma/surgery[MeSH Terms])
**Step 3**	AND ("radiotherapy"[Subheading] OR "radiotherapy"[All Fields] OR "radiotherapy"[MeSH Terms] OR "stereotactic body radiotherapy"[All Fields]))
**Step 4**	AND (low-risk[All Fields] OR early-stage[All Fields])
**Step 5**	AND (Clinical Trial[ptyp] OR Comparative Study[ptyp] OR Controlled Clinical Trial[ptyp] OR Journal Article[ptyp] OR Meta-Analysis[ptyp] OR Multicenter Study[ptyp] OR Observational Study[ptyp] OR "retrospective studies"[MeSH Terms] OR Pragmatic Clinical Trial[ptyp] OR Randomized Controlled Trial[ptyp] OR Review[ptyp] OR systematic[sb])
**Step 6**	AND (English[lang] OR French[lang])

The literature review identified 326 references. In the first stage of selection, we analyzed the titles of the articles; which led to the exclusion of 269 references. The second step of selection on abstract reading led to the exclusion of 39 references. The reasons for exclusion were related to: diagnosis of prostate cancer; treatment of other levels of risk for prostate cancer (intermediate orhigh) or other stages of pathology (metastatic); other treatments (active surveillance, brachytherapy, cryotherapy, focused ultrasound...) ; protocols for a clinical trial; quality of care (instead of patients' quality of life); guidelines on therapeutic strategies for the management of prostate cancer abroad ; to a case study; duplicates ; to a non-original article (expert opinion, response to author).

Finally, we used the same reasons for exclusion for the third stage of selection on full article reading. This allowed us to select 3 references to value transition probabilities and 2 references to enhance the utilities and quality of life data. The selection steps for references are detailed on a flowchart (figure 1).

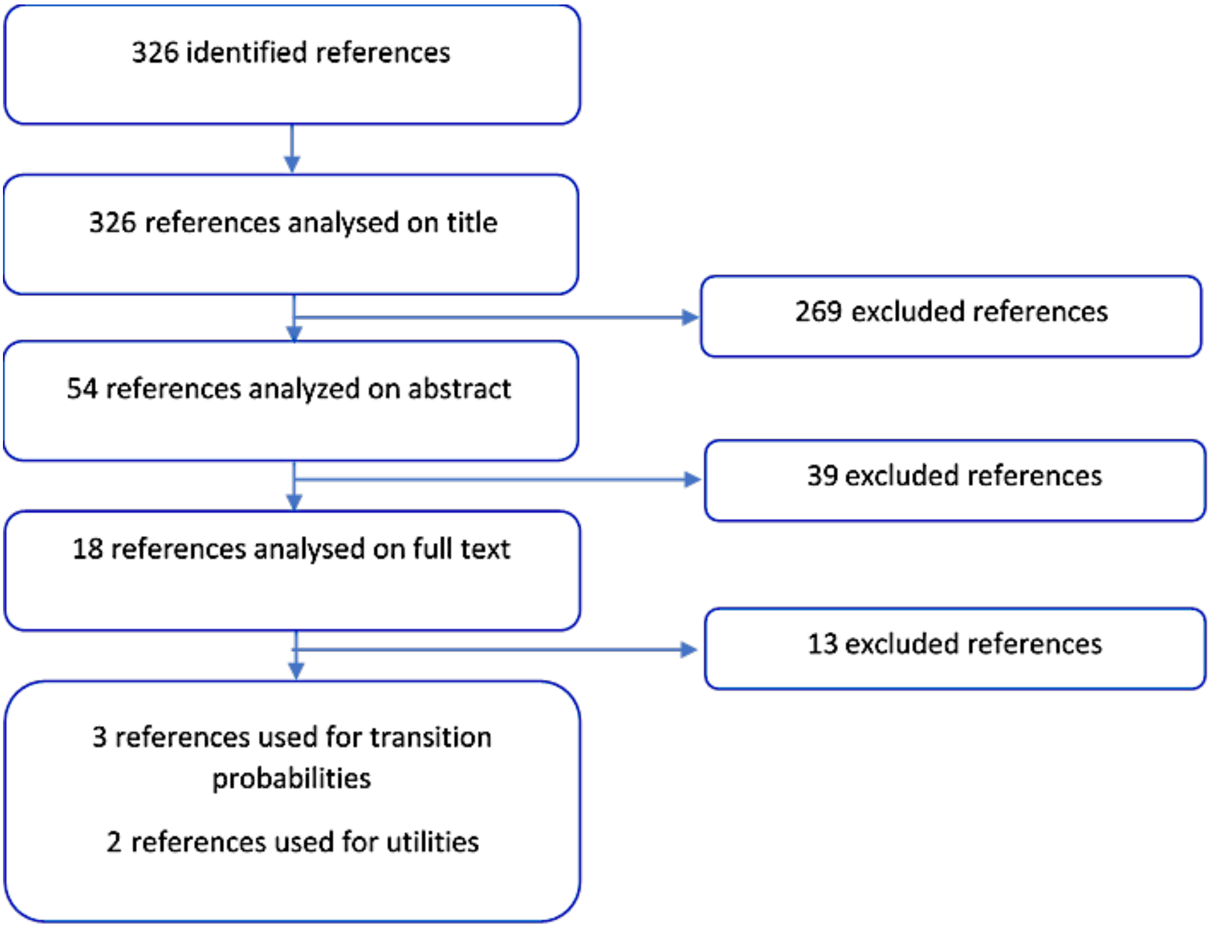

Figure 1 - Article selection flow chart

Elaboration of the decision model and assumptions

The path of care for patients with low-risk localized prostate cancer was determined from the French (1), (2) and European (3) guidelines and validated with the team of radiation oncologists.

In order to take into account the differential cost between the management up to the intervention (during the first year of treatment) and the differential cost during post-treatment follow-up (with different risks of toxicity depending on the interventions), a post-intervention state was created to integrate a notion of time (Figure 2).

In fact, patients only stay in the initial state during one cycle. Then they transit to the post-intervention state. This separates the costs associated with the robot-assisted intervention, which takes place in the first year, from the follow-up costs beyond the first year with consultations and complications related to the interventions.

If it was not found in the literature, the variation in parameters was set as follows: +/- 50% for transition probabilities variations, +/- 50% for costs, +/- 0,05 for utilities.

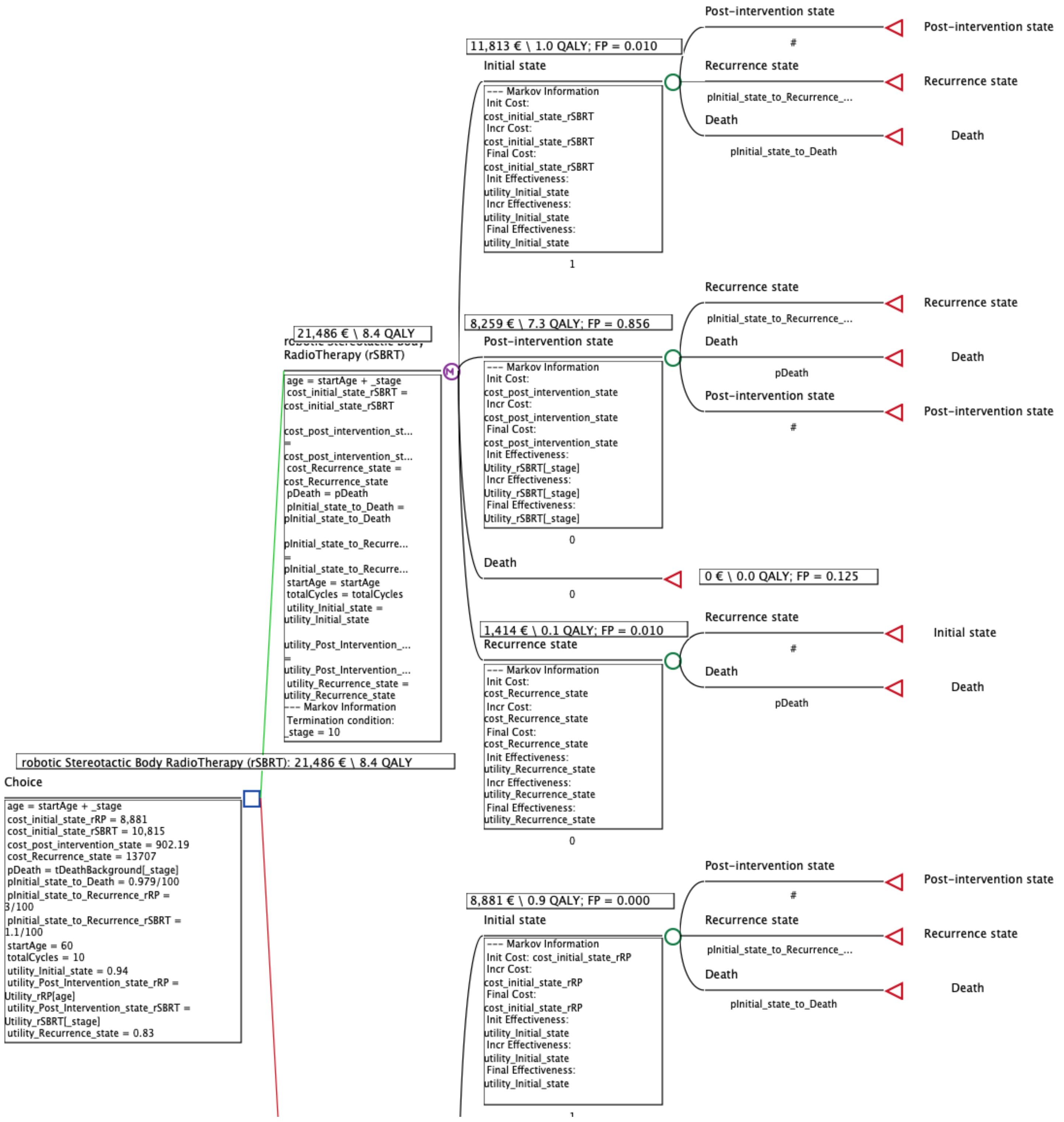
robotic Stereotactic Body RadioTherapy (rSBRT): 21,486 € \ 8.4 QALY
Choice
age = startAge + _stage
cost_initial_state_rRP = 8,881
cost_initial_state_rSBRT = 10,815
cost_post_intervention_state = 902.19
cost_Recurrence_state = 13707
pDeath = tDeathBackground[_stage]
pInitial_state_to_Death = 0.979/100
pInitial_state_to_Recurrence_rRP = 3/100
pInitial_state_to_Recurrence_rSBRT = 1.1/100
startAge = 60
totalCycles = 10
utility_Initial_state = 0.94
utility_Post_Intervention_state_rRP = Utility_rRP[age]
utility_Post_Intervention_state_rSBRT = Utility_rSBRT[_stage]
utility_Recurrence_state = 0.83
21,486 € \ 8.4 QALY
RadioTherapy (rSBRT)
age = startAge + _stage
cost_initial_state_rSBRT = cost_initial_state_rSBRT
cost_post_intervention_st... = cost_post_intervention_st...
cost_Recurrence_state = cost_Recurrence_state
pDeath = pDeath
pInitial_state_to_Death = pInitial_state_to_Death
pInitial_state_to_Recurre... = pInitial_state_to_Recurre...
startAge = startAge
totalCycles = totalCycles
utility_Initial_state = utility_Initial_state
utility_Post_Intervention_... = utility_Post_Intervention_...
utility_Recurrence_state = utility_Recurrence_state
--- Markov Information
Termination condition: _stage = 10
11,813 € \ 1.0 QALY; FP = 0.010
Initial state
--- Markov Information
Init Cost: cost_initial_state_rSBRT
Incr Cost: cost_initial_state_rSBRT
Final Cost: cost_initial_state_rSBRT
Init Effectiveness: utility_Initial_state
Incr Effectiveness: utility_Initial_state
Final Effectiveness: utility_Initial_state
1
Post-intervention state
#
Recurrence state
pInitial_state_to_Recurrence_...
Death
pInitial_state_to_Death
8,259 € \ 7.3 QALY; FP = 0.856
Post-intervention state
--- Markov Information
Init Cost: cost_post_intervention_state
Incr Cost: cost_post_intervention_state
Final Cost: cost_post_intervention_state
Init Effectiveness: Utility_rSBRT[_stage]
Incr Effectiveness: Utility_rSBRT[_stage]
Final Effectiveness: Utility_rSBRT[_stage]
0
Recurrence state
pInitial_state_to_Recurrence_...
Death
pDeath
Post-intervention state
#
Death
0
0 € \ 0.0 QALY; FP = 0.125
1,414 € \ 0.1 QALY; FP = 0.010
Recurrence state
--- Markov Information
Init Cost: cost_Recurrence_state
Incr Cost: cost_Recurrence_state
Final Cost: cost_Recurrence_state
Init Effectiveness: utility_Recurrence_state
Incr Effectiveness: utility_Recurrence_state
Final Effectiveness: utility_Recurrence_state
0
Recurrence state
#
Initial state
Death
pDeath
8,881 € \ 0.9 QALY; FP = 0.000
Initial state
--- Markov Information
Init Cost: cost_initial_state_rRP
Incr Cost: cost_initial_state_rRP
Final Cost: cost_initial_state_rRP
Init Effectiveness: utility_Initial_state
Incr Effectiveness: utility_Initial_state
Final Effectiveness: utility_Initial_state
Post-intervention state
#
Recurrence state
pInitial_state_to_Recurrence_...
Death
pInitial_state_to_Death

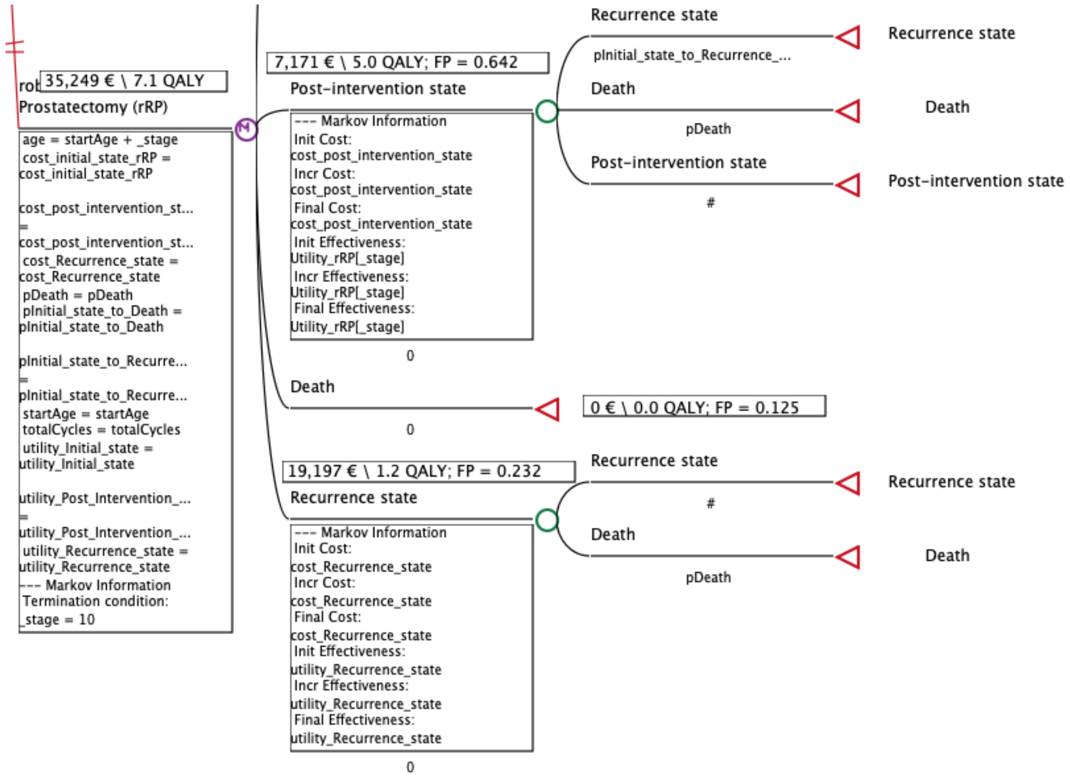

Figure 2: Markov Model comparing rSBRT and rRP

Markov model parameters

In order to achieve this model, it was necessary to determine the probability of transition between the health conditions of the model, the costs and outcomes associated with these states.

Transition probabilities

During each cycle, the transition probability estimates the proportion of patients who transit to another health state or remain in the same state. We conducted research in the literature and interviewed professionals to validate our hypotheses.

Our literature review identified the articles needed to calculate these parameters for patients with low-risk prostate cancer treated with prostatectomy or radiation therapy.

Regarding the transition probability to the "Death" state, we suggested that patients mortality in the initial state and in the post-intervention state after 1 year corresponds to mortality in the general French population that increases over time and does not differ between interventions (5).

Utilities

Localized prostate cancer has an impact on patients' quality of life. The utilities and references of these values, for initial state and recurrence state, were considered identical for both interventions, but not for the other state.

Costs

During a one-year cycle in the initial state, we considered that a patient benefited from one of the interventions and therefore we assessed the specific costs for each robot, performing procedures (imaging, anatomopathology), hospitalization for the prostatectomy group and outpatient sessions and transport for the robotic radiotherapy group. The risk of sexual, urinary and digestive toxicity following robot-assisted procedures were taken into account.

From a societal perspective, we collected and researched the costs of each comparative strategies. On the one hand, robot-assisted prostatectomies are coded as conventional prostatectomy acts. For private sector institutions, we have added the pricing of vesiculo-prostatectomy, curing and anatomopathological analysis (Table 2). On the other hand, for robotic stereotactic radiotherapy, there is a specific act valued at €1,346 per session, but only in the hospital sector (Table 3).

From a societal perspective, the cost of the initial state with robot-assisted prostatectomy (rRP) was estimated at €8,881 and with robot-assisted stereotactic radiotherapy at €10,815 (Table 3). In France, transport fees are refunded by the national health insurance after surgery or for SBRT. Depending on their condition, patients may have access to an ambulance but most of the time only specialized taxi rides called "VSL" are considered adequate to drive patients' home.

**Public hospitals**		**Private hospitals**	
Hospitalisation tariffs in public hospitals **(6)**	€8,721	Hospitalisation tariffs in private hospitals (6)	€6,281
		+ Code act JGFC001 : total vesiculo-prostatectomy by coelioscopy	+ €957
		+ Code act FCFC003 : lymphonodal curing	+ €273
		+ Code act JGQX004 : anatomopathological analysis	+ €146
Number of stays in public hospitals **(6)**	2,049	Number of stays in private hospitals (6)	893
**TOTAL**			
**Average cost of a prostatectomy in France (in public and private sectors)**	**€8,398**		

Table 2 - Details of the method of calculating the average cost of a prostatectomy stay (DRG 12C11 - Major pelvic interventions in humans for malignant tumours) in France from the average of the public and private hospitals according to the French national cost study in 2016 (6)

Compared interventions	rRP	rSBRT	Details and sources
**rRP cost**	€8,398	NA	Prostatectomy DRG (Disease related group) from National Cost Study [9] and calculation
**preparatory session before rSBRT**	NA	668€/ session<br>**€3,340/ 5 sessions**	For biopsy, we used the CCAM code ZZMK024 of "Preparation for external irradiation with scan tracking, three-dimensional dosimetry, virtual simulation using the beam's eye view function and three-dimensional rendition, and setting up a multilamemic collimator for intensity modulation"
**rSBRT cost**	NA	1347 € / session<br>**€6,735 / 5 sessions**	(7) (7)<br>For hospital-based rSBRT radiotherapy sessions, the Disease related Group of patients (DRG) code: 28Z11Z
**Other costs (consumables, side effects, monitoring, transport)**	€483	€740	**Interviews for additional material or equipment costs**: for rSBRT, 4 gold grains for fiducial technique at € 100 per unit (so €400/ 5 sessions) and, for rRP, the amount of € 1,450 is already included in DRG cost.<br>**Monitoring for the 1st year**: 1 specialized medical visit at €30 (7)<br>**Transports** (8)**:** 1 specialized taxi ride: €31/patient for rRP versus 5 round trips in specialized taxi : €310/patient for rSBRT<br>**Side effects**: Cost of thrombo-embolic event in prostate cancer (9), cost of bleeding with prostatectomy (10), probability of urinary complications (grade 3 or 4) for the rRP group (11), (12), for the rSBRT group, there were no grade 3 or 4 urinary side effects in the Katz et al. (13) (neither grade 3 or 4 rectal toxicity requiring surgery)
**Total cost per patient**	**€8,881**	**€10,815**	

Table 3 - Details of costs calculation to estimate the total cost of the initial state per patient for each intervention in societal perspective in France (radical robot-assisted prostatectomy or robot-assisted stereotactic radiotherapy) - NA : Not applicable

The costs of the other health states are detailed in table 4. The costs of the post-intervention state after 1 year was estimated at €902 and the cost of the recurrence state at €13,707 (14).

State	Cost /year/patient	Description and sources
**Post intervention state after one year**	€902	Average cost of conventional treatment for the entire cohort of Molinier *et al.* (14) after the first year of treatment up-to-date costs with the French inflation rate published annually by INSEE
**Recurence state**	€ 13,707	Average cost of recurrence state of prostate cancer patients according to the analysis conducted by Molinier *et al.* (14) which we have updated with the French inflation rate published annually by INSEE

Table 4 - Costs of post-intervention state and recurrence state for both interventions

References:

1. HAS I. Guide ALD Cancer de la prostate. (2012). https://www.has-sante.fr/upload/docs/application/pdf/2012-03/ald_30_guide__prostate_web.pdf#page17 [Accessed July 15, 2019]

2. Rozet F, Hennequin C, Beauval J-B, Beuzeboc P, Cormier L, Fromont G, Mongiat-Artus P, Ouzzane A, Ploussard G, Azria D, et al. Recommandations en onco-urologie 2016-2018 du CCAFU : Cancer de la prostate. *Progrès en Urologie* (2016) **27**:S95–S143. doi: 10.1016/S1166-7087(16)30705-9

3. Mottet N, Bellmunt J, Bolla M, Briers E, Cumberbatch MG, De Santis M, Fossati N, Gross T, Henry AM, Joniau S, et al. EAU-ESTRO-SIOG Guidelines on Prostate Cancer. Part 1: Screening, Diagnosis, and Local Treatment with Curative Intent. *Eur Urol* (2017) **71**:618–629. doi: 10.1016/j.eururo.2016.08.003

4. Hamdy FC, Donovan JL, Lane JA, Mason M, Metcalfe C, Holding P, Davis M, Peters TJ, Turner EL, Martin RM, et al. 10-Year Outcomes after Monitoring, Surgery, or Radiotherapy for Localized Prostate Cancer. *N Engl J Med* (2016) **375**:1415–1424. doi: 10.1056/NEJMoa1606220

5. Arvold ND, Chen M-H, Moul JW, Moran BJ, Dosoretz DE, Bañez LL, Katin MJ, Braccioforte MH, D'Amico AV. Risk of death from prostate cancer after radical prostatectomy or brachytherapy in men with low or intermediate risk disease. *J Urol* (2011) **186**:91–96. doi: 10.1016/j.juro.2011.03.003

6. ENC MCO | Stats ATIH. https://www.scansante.fr/applications/enc-mco/submit?snatnav=&annee=2016&secteur=dgf&type_activite=ghs&cmd=&souscmd=&racine=12C11&ghm=12C11&mbout=dummy&num_selection=12C11&type_selection=racine&zip=non [Accessed August 26, 2019]

7. AMELI. Tarifs des consultations françaises. https://www.ameli.fr/assure/remboursements/rembourse/consultations/modifications-tarifs-consultations-mai-2017 [Accessed July 26, 2019]

8. Cour des comptes. Rapport_securite_sociale_2012 - Chapitre XI - Les transports de patients à la charge de l'assurance maladie. (2012). https://www.ccomptes.fr/sites/default/files/EzPublish/rapport_securite_sociale_2012_transports_patients.pdf [Accessed July 26, 2019]

9. Scotte F, Martelli N, Vainchtock A, Borget I. The Cost of Thromboembolic Events in Hospitalized Patients with Breast or Prostate Cancer in France. *Adv Ther* (2015) **32**:138–147. doi: 10.1007/s12325-015-0187-3

10. Haute Autorité de Santé CA. Évaluation des dimensions clinique et organisationnelle de la chirurgie robot-assistée dans le cadre d'une prostatectomie totale. (2016). https://www.has-sante.fr/upload/docs/application/pdf/2016-12/rapport_robot_vd.pdf [Accessed July 13, 2019]

11. Yaxley JW, Coughlin GD, Chambers SK, Occhipinti S, Samaratunga H, Zajdlewicz L, Dunglison N, Carter R, Williams S, Payton DJ, et al. Robot-assisted laparoscopic prostatectomy versus open radical retropubic prostatectomy: early outcomes from a randomised controlled phase 3 study. *Lancet* (2016) **388**:1057–1066. doi: 10.1016/S0140-6736(16)30592-X

12. Wallerstedt A, Tyritzis SI, Thorsteinsdottir T, Carlsson S, Stranne J, Gustafsson O, Hugosson J, Bjartell A, Wilderäng U, Wiklund NP, et al. Short-term Results after Robot-assisted Laparoscopic Radical Prostatectomy Compared to Open Radical Prostatectomy. *European Urology* (2015) **67**:660–670. doi: 10.1016/j.eururo.2014.09.036

13. Katz AJ, Kang J. Quality of Life and Toxicity after SBRT for Organ-Confined Prostate Cancer, a 7-Year Study. *Front Oncol* (2014) **4**:301. doi: 10.3389/fonc.2014.00301

14. Molinier L, Castelli C, Bauvin E, Rebillard X, Soulié M, Daurès J-P, Grosclaude P. Cost study of the clinical management of prostate cancer in France: results on the basis of population-based data. *Eur J Health Econ* (2011) **12**:363–371. doi: 10.1007/s10198-010-0250-6
